# Supplementary material for: Are carbon emissions trading and green financial instruments synergistic? -Comprehensive quantitative research based on content analysis
Source: PLoS One. 2024 Mar 7;19(3):e0298601. doi: 10.1371/journal.pone.0298601 (PMC10919601; doi:10.1371/journal.pone.0298601)
Supplement: S1 Data — (ZIP) [file pone.0298601.s001.zip › Data/地级市Z_F工作报告.zip!/░▓╗╒/╬▀║■╩╨/└┤╫╘╣1⁄2╓┌║┼í╛┬φ┐╦╩2╛▌═°í┐_2012.docx]

——2012年1月10日在芜湖市第十四届人民代表大会第六次会议上

市长 杨敬农

各位代表：

现在，我代表市人民政府，向大会作政府工作报告，请各位代表审议，并请市政协委员和其他列席人员提出意见。

一、“十二五”发展实现良好开局

2011年是“十二五”发展的开局之年，也是行政区划实现历史性重大调整之年。在中共芜湖市委的坚强领导下，全市人民牢牢把握科学发展的主题和全面转型、率先崛起、富民强市的主线，凝心聚力，务实奋进，努力推动经济社会又好又快发展，顺利完成了市十四届人大五次会议确定的各项目标任务，阔步迈入两岸融合、拥江发展的新时代。

初步预计，全市实现地区生产总值1600亿元，比上年增长16%；财政收入286.8亿元，其中地方财政收入140亿元，分别增长31.3%和33.4%；全社会固定资产投资1330亿元，增长27%；全部工业销售收入3650亿元，增长35 %；社会消费品零售总额418亿元，增长18%；进出口总额39.6亿美元，增长46.1%；城市居民人均可支配收入21500元，农民人均纯收入8280元，分别增长15%和16%；城镇化率提高2个百分点；高新技术产业增加值占地区生产总值比重提高2个百分点；第三产业增加值增长10%；新兴产业实现增加值170亿元，增长26%；城镇新增就业8.5万人，城镇登记失业率3.3%；人口出生率控制在8.7‰以内；完成省政府下达的节能减排任务。

一年来，我们主要做了以下工作：

（一）积极采取有效措施，经济保持平稳快速发展。面对错综复杂的国内外形势，我们深入研究，未雨绸缪，积极应对。一是谋划和完善政策措施。出台了支持自主创新及战略性新兴产业、促进房地产市场健康发展、鼓励企业上台阶、扶持中小企业和微型企业等政策，各级财政兑付支持资金52亿元。安排39户“小巨人”企业奖补资金5000万元。拨付家电下乡、家电以旧换新补贴资金1.6亿元。市级调度资金162亿元，支持县区经济发展。我市成为国家开展企业股权和分红激励试点城市、全国老工业基地规划改造城市，高新区“新三板”试点获证监会备案。二是积极拓宽融资渠道。顺荣股份、亚夏汽车公司成功上市，全市企业在资本市场融资159亿元。融资性担保机构为中小企业贷款担保额125亿元。委托银行向中小企业发放贷款7亿元。年末社会融资总量余额2616亿元，比年初增长23.6%。三是加快推进重大项目建设。全年在建投资亿元以上非房地产项目450个，总投资2500亿元，其中10亿元以上项目59个。奇瑞整车改扩建、芜湖海螺水泥三期、美的日电基地、富鑫特钢二期、响水涧抽水蓄能电站1号机组等项目建成投产，奇瑞重工、格力电器、双钱轮胎、德力西电气、南翔物流等项目开工建设。宁安铁路、合福铁路、芜雁高速、芜申运河工程加快建设，清水河西幅公路桥建成通车，芜湖长江公路二桥获批立项，芜湖电厂五期2号机组项目获得核准。长江干线芜湖长江大桥以下航道维护水深最高提升至10.5米，裕溪口水道开通为国家公用航道。四是强化企业服务。坚持深入企业调研，破解发展难题。对1941家企业开展了政策培训，帮助企业广泛知晓政策、充分利用政策。出台22项企业用工服务举措，开展“春风行动”、校企对接等大型招聘会200场次。办理企业税收优惠49.5亿元，其中及时办理出口退税18亿元；减缴社保费3.1亿元，缓缴社保费2.9亿元，发放社保补贴和岗位补贴8938万元，切实减轻企业负担。

（二）促进产业高端化发展，经济结构日趋优化。汽车及零部件、材料、电子电器、电线电缆四个支柱产业实现总产值2500亿元，增长40%。战略性新兴产业完成投资200亿元，增长40%。现代服务业培育取得新进展，发展后劲进一步增强。南陵农村商业银行开业运营，华夏通第三方支付牌照获批，组建了注册资金均为30亿元的皖江金融租赁公司、安徽新安金融集团，新增小额贷款公司22家、证券期货机构4家、典当行3家、担保公司4家、股权投资基金5只，金融服务中心开工建设。裕溪口煤炭储配中心一期、安得物流东区物流中心、瑞丰商品博览城一期、果品冻品水产批发市场等建成运营。新建万吨级码头1个、5000吨级泊位5个。镜湖万达广场、苏宁广场等大型商业综合体开工建设。新增文化创意企业20家、服务外包企业40家，服务外包收入超1亿美元，我市成为省服务外包基地城市。新建成开业和在建的4星级标准以上酒店10家，新开业快捷酒店22家、5000平方米以上大型超市6家。方特欢乐世界、梦幻王国共接待游客365万人次，增长74.3%。大浦农业自然灾害教育体验主题公园建成运营。举办了第三届中国国际动漫创意产业交易会等18个大型会展。现代农业发展取得新成效，粮食生产喜获丰收，205国道沿线现代农业示范产业带、5000亩蔬菜标准园和蔬菜直销店建成，南陵蓝莓种植基地被批准为全国农业标准化示范区，推广种植超级稻50万亩，新增无公害、绿色食品20个，年销售收入亿元以上产业化龙头企业达25家，我市成为全国农产品现代流通综合试点市。

（三）推进自主创新，转型发展内生动力增强。全市新增认定和公示的高新技术企业47家、高新技术产品133个，争取国家级和省级重点科技项目107项。芜湖节能与新能源汽车产业成为全国首批创新型产业集群试点，奇瑞公司成为首批国家级技术创新示范企业，华东光电技术研究所成为国家级创新型企业。新增科技企业孵化器5.35万平方米、“科技路路通”创新服务站12家。精诚铜业技术中心被认定为国家级企业技术中心，新增省级研发机构29家。专利申请量9500件、授权量6500件，分别增长19.3%和60.3%，3个县跻身全省专利申请总量十强县，4家企业进入全省发明专利申请十强企业。商标注册核准量1634件，鲁班建筑、欣菱电缆、神剑新材料、铁扇灭火设备、安得物流被认定为“中国驰名商标”，四季春、老余昌被认定为“中华老字号”，南陵大米被批准为“国家地理标志保护产品”，新增安徽名牌产品15个。出台政府质量奖管理办法，启动首届政府质量奖评价工作，初步建成农产品质量检测网络体系。实施技术标准发展战略，承担国家标准、行业标准制定计划27项，制定省地方标准16项。举办产学研合作对接活动19次，与中国科大、浙江大学等高校院所深化科技合作。培养各类人才3.5万人，新增高端人才1500人、产业创新团队11个。我市第五次蝉联全国科技进步先进市，成为十城万盏半导体照明应用工程试点示范城市，3个区被评为全国科普示范区。

（四）统筹城乡发展，城市功能不断完善。启动了新一轮城市总体规划、全市空间发展战略规划、土地利用总体规划、产业发展与布局规划的修编，完成了银湖、凤鸣湖、荆山等地区控制性详细规划。城市基础设施建设完成投资135亿元。东区纬四路、三山区龙湖路二期、赤铸山东路跨扁担河桥、中江大道跨青弋江大桥建成通车，完成了花津桥和赭山东路、216省道改造工程。白马山路、峨溪路、利民东路、黄山东路延伸段、长江南路二期、峨山路东延立交、中江大道南延伸段等干道以及利民路水厂二期、三山水厂项目开工建设。城乡电网建设完成投资6.1亿元。积极争创国家园林城市，西洋湖等10个城市公园加快建设，新增绿地面积450万平方米。举办了首届“刘开渠奖”国际雕塑大展，芜湖雕塑公园建成开放。完成了68个老旧住宅小区整治、17条城市道路杆线下地工程，改造节能路灯1.3万盏，实施亮化项目24个，新增公交车378台。三荻路青弋江桥建成通车，无为县通江大道等5条公路列入省道规划，危桥改造竣工21座，新改建农村公路132公里，改造农村危房2532户，完成了5座水库除险加固、3条河流治理、18.84万农村人口饮水安全工程，农村生产生活条件进一步改善。完成房地产投资300亿元，增长20%；商品房价格理性回归，房地产市场保持稳定。强化城市管理，坚持“市长上路”制度，解决突出问题。开展市容整治提升年活动，数字化城管平台覆盖面由72平方公里扩大到260平方公里，对弋矶山医院广场、保兴垾、长江沿岸等重点地段进行专项整治。坚持节约集约利用土地，竣工标准化厂房64万平方米，盘活工业用地7167亩。着力保护生态环境，启动了板城垾水系改造，完成19个住宅小区雨污分流改造、11项农村清洁工程。实施69个减排重点项目，滨江污水处理厂建成运行，开展电厂脱硝改造、水泥企业实施低氮燃烧改造和农业污染源治理，停产整治涉铅重金属企业6家，整合关闭非煤矿山20家。完成植树造林2.1万亩，建成抑螺防病林6000亩。南陵县成为国家级生态示范区，5个镇成为“全国环境优美乡镇”。

（五）深入推进改革开放，发展活力持续迸发。深化医药卫生体制改革，巩固基层综合医改成效，启动县级医院综合改革。稳步推进公立医院改革试点，深入推进医药分开，20家公立医院率先实行药品“零加成”销售，基本药物制度和药品供应保障体系初步建立。推进统筹城乡综合配套改革，建设土地承包经营权流转服务中心、农村土地流转有形市场。大力实施土地整治，新增耕地15480亩，完成城乡建设用地增减挂钩（置换）指标6240亩。民营经济健康发展，入围全省民营企业百强和出口创汇百强的企业有60家，居全省第一。创新财政管理，加强土地出让金征缴管理，提高资金入库率和使用效益；将政府性资金存放银行与信贷投放挂钩，引导和激励银行加大信贷支持力度；建立了偿债准备金制度，提高政府性债务的风险防范能力；开展了市直机关会计代理核算，把好预算资金出口关和结算关，提高财政资金效益。积极推进皖江城市带承接产业转移示范区建设，扩大对外开放。坚持挑商选资，实施精准招商，在苏州、香港等地举办了10场投资环境推介会，全年实际利用内资1420亿元，实际利用外资10.4亿美元，分别增长28.9%和26%。新增7家境外世界500强企业来芜投资，进出口备案登记企业增加235家。完成港口吞吐量7300万吨、集装箱21万标箱，均创历史新高。加强区域合作，与马鞍山市签订同城化建设合作框架协议，亳州芜湖工业园区等项目启动建设，出资8000万元援建的亳州华佗技师学院建成使用。外事、侨务、对台、外经等工作不断加强。

（六）大力保障和改善民生，人民生活水平进一步提高。投入47亿元，实施了39项民生工程。出台了关于实施富民行动的意见，努力促进城乡居民收入持续提高。落实各项就业政策，完成农民工技能培训1.3万人、新型农民培训2万人；新增发放就业再就业小额担保贷款3.5亿元，累计8.5亿元，居全省第一位。完善社会保障体系，城镇职工养老、失业、医疗、工伤、生育保险参保总数196万人次，增长17.5%；四县全部纳入国家新型农村养老保险和城镇居民养老保险试点，39.9万人享受养老保障。城乡居民医疗保险基本实现全覆盖，新型农村合作医疗参保219万人、参合率98%以上，建立了新型农村合作医疗、城镇居民医疗保险与城镇职工医疗保障转接机制，工伤保险实现市级统筹。全市退休人员养老金月均增加133元，失业保险金月均增加78元，市区最低工资标准提高到800元。发放低保金3.5亿元，保障了16万城乡低保对象基本生活。向城乡低保对象和五保户发放价格临时补贴4350万元。实施困难群众医疗救助、临时救助和重度残疾人、重大传染病病人生活救助4.5万人次，支出救助金8000多万元。新改建敬老院、福利院27所。新建和改造市区菜市场10处。努力提高住房保障水平，对人均住房建筑面积15平方米以下的城镇低收入家庭实现应保尽保，实物配租3610户，发放租金补贴14758户；财政投入22.5亿元，建设保障性住房5.6万套；扩大住房公积金制度覆盖面，新增缴存职工5万人，发放住房公积金贷款8.5亿元。

（七）加大公共服务投入，社会事业全面进步。文明创建活动取得积极成效，我市成为第二届全省文明城市，芜湖县城、繁昌县城成为全国文明县城。实施学前教育三年行动计划，新建公办幼儿园9所，扶持普惠性幼儿园70所，学前三年毛入园率达75%。投资6.1亿元，新建、改扩建学校59所。投资1.2亿元，实施中小学抗震、加固、重建校舍22.4万平方米。市一中新校区、安徽中加学校投入使用。建成230个农村留守儿童之家。完成121所义务教育学校标准化建设。免除义务教育学杂费、教科书费和补助贫困寄宿生生活费共计6074万元，补助公用经费2亿元。保障外来就业人员子女平等接受义务教育，残疾儿童少年入学率达98%。实施高中招生改革，高中阶段毛入学率达86.5%，市区51%学生进入省示范高中学习。资助普高和中职学校家庭困难学生2.2万名，发放助学金3281万元。推进职教园区建设，工业学校、师范学校新校区开工，6所民办学校进入园区办学。首个民办高校——安徽扬子职业技术学院建成招生。完善城乡卫生服务体系，建成12个标准化村卫生室，规范化建设17个社区卫生服务机构，健全了公立医院与基层医疗卫生机构的分工协作机制。搭建卫生信息平台，推进居民电子健康档案建设。加强人口和计划生育工作，利益导向机制进一步完善。城市规划馆和博物馆开工建设，建成了4个县区图书馆和文化馆、23个乡镇综合文化站、356个农家书屋。全市有4个文化馆被评为国家一级馆，图书馆、文化馆全部免费向社会开放。加强文物保护，实施了圣雅各教堂保护性平移，开工建设范罗山民间收藏博览园。开展了庆祝中国共产党成立90周年系列文艺活动。我市再次被评为全国文化体制改革先进地区，镜湖区被命名为“中国民间文化艺术之乡”。广泛开展全民健身活动，60%中小学校体育设施免费向群众开放。承办了全国青少年车辆模型教育总决赛、全国青少年建筑模型教育总决赛、全国女子手枪射击邀请赛、足协杯上海申花队主场比赛等赛事。我市运动员在国际和全国重大体育赛事中，夺得奖牌14枚。强化安全生产“一岗双责”制，公开挂牌整治91个重大隐患，安全生产保持平稳向好态势。推进依法治市，启动了“六五”普法，创建民主法治示范村（社区）37个，完成了第八届村委会换届选举。认真做好优抚安置工作，加强国防后备力量建设。强化信访责任制度，集中研究解决重大积案，信访形势持续好转。加强社会治安综合治理，着力排查化解矛盾纠纷，严厉打击违法犯罪，保持了社会和谐稳定。人民防空、民族、宗教、气象、档案、地方志、妇女儿童、老龄、残疾人等事业取得新进展。

（八）强化政府管理，效能建设深入开展。自觉接受市人大及其常委会的法律监督和工作监督、市政协的民主监督，主动加强与各民主党派、工商联、无党派人士的联系，办理人大议案2件、建议124件和政协提案325件，回复网民咨询和市长信箱留言8700余条。公开招考录用公务员217名，公开招聘录用事业单位工作人员276名。推行建设项目“并联审批”、企业注册登记“一表通”、收费管理“一票制”等模式，对重点投资和建设项目实行行政审批“直通车”服务。强化招投标工作精细化管理，完成政府性招标采购项目3442项。取消行政事业性收费项目14项。坚持依法行政，对行政处罚工作进行了全面检查，规范行政处罚自由裁量权。开展了“大走访”、以“改进作风，优化环境”为主题的机关效能建设活动，加快转变机关作风和党员干部工作作风。积极推进行政管理和公共服务标准化，市政府成为国家级服务业标准化试点单位。严格落实廉政建设责任制，开展了廉政风险防控管理试点，加大对财政资金、建设资金、土地出让资金的审计监督力度，强化对权力运行的公开和监督，严肃查处违法违纪行为，政务环境进一步改善。

各位代表！

这些成绩，是在宏观环境复杂严峻、制约发展因素增多的情况下取得的，确实来之不易。这是中共芜湖市委正确领导、科学决策的结果，是人大代表充分行使职权、政协委员积极参政议政的结果，是全市人民团结一致、奋力拼搏的结果。在此，我谨代表市人民政府，向全体人大代表、政协委员，向全市广大工人、农民、知识分子、干部、驻芜解放军指战员、武警官兵和政法干警，向各民主党派、工商联、各人民团体、无党派人士、离退休老同志和社会各界人士，向在我市创业的投资者、建设者，向关心、支持芜湖发展的中央、省直各部门和海内外友好人士，表示崇高的敬意和诚挚的感谢！

各位代表！

我们清醒地看到，芜湖发展势头强劲，但仍存在诸多矛盾和挑战。经济规模不够大，战略性新兴产业培育任重道远，服务业发展相对滞后；土地、资源、环境制约日益突出，节能减排任务十分艰巨；社会建设和管理需要进一步加强，城乡居民收入、民生保障水平有待提高，不少群众生活还比较困难；政府职能转变还不到位，一些政府工作人员思想观念、能力素质、工作作风还不适应形势发展要求，一些领域腐败现象依然存在。对此，我们一定高度重视，坚决采取有力措施加以解决，绝不辜负全市人民的期望和重托！

二、2012年工作的总体要求和主要目标

今年是本届政府任期的最后一年，也是深入实施“十二五”规划的关键之年。当前，经济全球化、区域经济一体化深入发展，和平与发展仍是当今世界的主流，但国际金融市场剧烈动荡，世界经济形势总体上将更加严峻复杂，发达国家经济复苏的不稳定性不确定性上升。我国保持经济较快增长、社会和谐稳定，但经济增长下行压力和物价上涨压力并存，节能减排形势更趋严峻，部分企业生产经营困难加重，区域竞争更加激烈，经济金融等领域也存在诸多不容忽视的潜在风险。我们越是在发展好、发展快的时候，越是要保持更加清醒的头脑；越是在面对严峻的形势和挑战的时候，越是要更加坚定信心、沉着应对。我们应该看到，经过改革开放30多年特别是“十一五”的发展，我们集聚了巨大能量，积累了丰富经验，我市经济社会发展的基石更加稳固，产业发展多点支撑的基础更加坚实，国家支持产业转移、自主创新等政策叠加效应日益显现，区划调整后城市发展的势能显著增强，我们完全有条件、有能力、有信心应对各种挑战、化解各种风险，实现经济社会发展稳中求好、好中求快。

做好2012年政府工作，要全面贯彻党的十七届五中、六中全会精神和省、市第九次党代会战略部署，认真落实中央和省经济工作会议要求，坚持以科学发展为主题，以全面转型、率先崛起、富民强市为主线，紧紧围绕建设创新、优美、和谐、幸福新芜湖的目标，坚定不移地推进创新驱动、产业强市、统筹城乡、开放合作、和谐发展战略，更加突出改革开放，更加突出转型发展，更加突出富民导向，更加突出生态文明，更加突出文化繁荣，努力保持经济社会持续又好又快发展。

综合各方面因素，今年经济社会发展的主要目标是：地区生产总值增长14%，财政收入与经济保持同步增长，全社会固定资产投资增长20%，全部工业销售收入增长23 %，社会消费品零售总额增长17%，进出口总额增长15%，城市居民人均可支配收入、农民人均纯收入均增长15%，城镇化率提高2个百分点，高新技术产业增加值占地区生产总值比重提高2个百分点，第三产业增加值增长11%，新兴产业实现产值增长40%，城镇新增就业7万人，城镇登记失业率控制在4.5%以内，人口出生率控制在11‰以内，节能减排完成省政府下达任务。

实现上述目标，我们必须把中央精神和省委、省政府要求与芜湖实际紧密结合起来，切实增强工作的前瞻性、针对性和有效性，创造性地开展工作，力争完成得更好。

三、全力推动经济转型发展

加速芜湖崛起进程，必须切实把全面转型贯穿于经济社会发展的全过程。我们要紧紧抓住全球创新集聚爆发、新兴产业加速成长的战略机遇，坚持在加快发展中主动转型，在主动转型中加快发展。

（一）加快建设创新型城市

完善政策体系，加大投入力度，强化工作措施，全面推进国家技术创新工程试点和合芜蚌自主创新综合试验区建设。深入实施人才强市战略，出台企业股权和分红激励试点实施办法，先行先试，力求突破；突出创新创业领军人才、高技能人才两个重点，强力实施领军型科技创业人才引进计划。完善创业中心、大学科技园等科技创新平台和载体，新建省级以上研发机构20家，新增科技企业孵化器5万平方米。建立企业主导的产学研合作新模式，创新产学研人员双向流动合作新机制，引进高校院所研发机构2家以上。争取新申报认定高新技术企业30家、高新技术产品100个。力争专利申请量1万件、授权量4000件，促进专利技术产业化。争创国家商标战略实施示范城市和全国知名品牌创建示范区，新增中国驰名商标和省著名商标25件、安徽名牌产品15个。力争承担制定发布国家标准和行业标准5项、省级地方标准8项。积极筹建国家特种电缆质量监督检验中心，加快建设3个省级质量监督检验中心。积极推进国家科技和金融结合试点、创新型产业集群试点。建设高新技术企业产权交易平台，打造区域股权私募融资市场。推进高新区“新三板”试点，争取首批8户企业挂牌，支持高新技术中小企业发行集合债。

（二）着力提升产业层次

集中有限人力物力财力，聚焦突破，着力发展先进制造业。壮大汽车及零部件、材料、电子电器、电线电缆四个支柱产业，完善产业链配套，加速集群化发展，推动产业不断升级。加快培育和发展高端装备、光电光伏、新材料、智能家电四个战略性新兴产业，完善产业发展指导目录，制定产业链建设计划，做大产业群，提升竞争力。发挥奇瑞、海螺、新兴铸管等大企业的优势，打造汽车及高端装备基地。积极推动信息化与工业化融合，运用高新技术和先进适用技术改造提升传统产业。

把加快服务业发展作为调整经济结构的战略重点，作为经济工作的重大任务。进一步完善服务业发展扶持政策、规划目标和考核体系，优先发展金融、现代物流、文化创意、服务外包、旅游五大服务业，大力发展总部经济。积极引进、培育法人金融机构，推进金融创新，建设皖江金融中心。着力破解企业融资难，引导支持域内外银行加大信贷投放，加快企业上市、发债进程，鼓励企业通过股权出质出资、动产抵押、知识产权质押、打捆贷款、出口退税质押等方式融资。将民强担保公司注册资本金由5亿元提高到20亿元，成立担保集团，着力解决中小企业融资难问题。大力支持小额贷款、担保、典当、股权基金等企业规范发展。推进江北港口规划建设，加快实施芜湖港、南翔万商、安得物流等重点项目，建设现代物流中心。推进服务外包产业园等项目，建设国家级服务外包基地城市。办好第五届中国科普产品博览交易会等重点会展。发展科技咨询、中介服务、商贸、养老、家庭服务和会展业，鼓励规模化、品牌化、网络化经营。

用工业化和生态化理念引领农业，促进农业现代化。落实好强农惠农政策，增加“三农”投入。加强农业示范园区建设，开展水稻、棉花、油菜高产创建活动，推广袁隆平超级稻种植，创建1万亩蔬菜标准园，深入实施畜牧业升级计划、水产跨越工程，提升农业综合生产能力。加快土地承包经营权流转步伐，大力引进辐射带动能力强的产业化龙头企业，规范发展农民专业合作经济组织，培育家庭农场、种养大户等现代农业经营主体，健全农业社会化服务体系，不断提高农业规模化、组织化水平。

坚持把扩大有效投资作为重中之重的长期任务，把项目建设作为经济工作的生命线，坚持市领导分工联系项目制度，加快华谊化工、奇瑞重工、格力电器、中软芜湖软件园等526个亿元以上非房地产开发重点项目建设。全面落实国家支持实体经济发展的财税政策，完善 “小巨人”企业培育计划等扶持政策，支持大企业提升竞争力，扶持中小企业、微型企业加速成长。

（三）统筹推进城市现代化

加快推进两岸一体化发展，整体提升江南、加快建设江北，彰显秀美山水，突出人文特色，提升城市品质。坚持规划引领，高起点修编城市总体规划和综合交通、商业网点等专业专项规划，完善四县四区战略发展规划，统筹裕溪口、沈巷、二坝等地规划，打造芜湖江北新城，切实做好城市空间设计、环境设计和形象设计。

推进城市大建设，安排投资150亿元，进一步完善城市基础设施。建成芜雁高速公路芜湖段，加快宁安铁路、合福铁路、芜申运河建设，开工建设长江公路二桥、无六路改建、芜湖火车站改扩建和站区枢纽工程，加快商合杭铁路、北沿江高速、铜南宣高速、芜黄高速、铜巢高速公路等项目前期工作。加快干线公路网建设，建成三荻路漳河桥、马园河桥等工程，开工建设205国道拓宽升级、梦溪路、鸠江北路、弋江路北延及快速化改造、中江桥改建和城南综合客运站等项目，续建峨山路东延伸段、中江大道南延伸段、黄山东路延伸段、长江路北延伸段、长江南路等道路。大力推进市区和县城的老城区改造，加快拆迁安置房建设。加强和改善房地产市场调控，保持房地产市场健康稳定发展。优先发展公共交通，大力实施道路畅通工程，再建一批人行天桥、公共停车场、公交港湾，新增293台公交车。建成利民路水厂二期工程。建设9个110千伏以上输变电工程项目。实施城市信息化工程，建设“智慧城市”。

加快新农村建设。完善镇村布点规划，推进城市基础设施和公共服务向农村延伸覆盖。确定一批市级新农村建设试点村，整合各种涉农资金集中投入，以土地整治整村推进为抓手，实施村庄整治，改造农村危房，推进农村清洁工程。抓住水利发展的难得机遇，加快青弋江分洪道工程建设，强力推进裕溪河及西河综合治理、青弋江干流加固、长江河道整治等重大项目前期工作，组织实施农村安全饮水、病险水库除险加固、中小河流治理、联圩并圩以及无为县万亩以上圩口堤防达标建设，全面开展农村沟渠湖塘清淤疏浚，推进小型农田水利重点县建设和建管体制改革。加快县乡道路升级改造、危桥改造、渡改桥和农村公路工程建设。加大无为西部山区和革命老区等地扶贫开发力度。

强化城市管理。开展“市容整治规范年”活动，加强责任区网格化管理，严格控制和坚决查处违法建设，推进城市管理信息化、规范化、市场化和城乡环卫工作一体化，提高城市管理水平。

（四）扎实推进生态文明建设

严格落实节能减排目标责任制，健全激励和约束机制。坚决抑制高耗能产业过快增长，突出抓好工业、建筑、交通、公共机构等领域节能，推进重点节能项目建设。大力发展新能源，申报建设国家新能源示范城市。实施主要污染物排放总量控制，加强重点行业、重点企业污染防治。加快城镇集中式污水处理设施和配套管网建设，强化污水处理设施运行管理和污水处理厂提标改造，加快老旧住宅小区雨污分流改造，建成城东污水处理厂，开工建设长江大桥开发区、二坝两个污水处理厂。完成垃圾无害化焚烧厂扩能改造。推动燃煤发电机组脱硝设施建设和水泥行业低氮燃烧改造。加强农业面源、机动车尾气污染治理。

着力提高资源节约集约利用水平。提高项目准入门槛，严把土地投资强度、环保、节能、产业布局、亩均税收五个关口，严控镇村工业园区建设。实行最严格的耕地保护政策，完善土地管理责任制，加大工业用地和经营性用地清理力度，加大土地使用税征收和奖励力度，对低效用地坚决“腾笼换鸟”，盘活存量，提高使用效益。鼓励建设多层标准化厂房，完成100万平方米建设任务。统筹规划利用长江岸线资源，坚决制止乱占乱建。规范矿产资源开采、矿业权出让转让行为，关停整合小矿山。编制循环经济产业园区建设规划，推进循环经济产业园示范基地建设，加快再制造产业发展，开工建设餐厨废弃物资源化利用和无害化处理工程项目。

巩固创建国家园林城市成果，启动国家生态园林城市创建工作，健全生态文明建设指标体系和考评机制。大力开展植树造林，推进城乡道路绿化，推广立体绿化，完成神山公园改造一期工程，加快大阳垾湿地公园等10座城市公园建设。加大水环境保护力度，加强银湖、凤鸣湖、龙窝湖、板城垾污染治理，启动保兴垾上游及支流整治。

（五）继续深化改革开放

深化重点领域和关键环节改革。一是深化医药卫生体制改革。积极稳妥推进公立医院改革试点，深入推进县级公立医院综合改革，健全三大医疗集团和公立医院的薪酬激励约束机制。完善公立医院药品“零加成”销售制度，建立医药分开和医用耗材集中采购的长效机制，探索大型医用设备统一管理新模式。二是深化国企改革。加强基金、小额贷款、担保等机构的国有股权管理，健全国有资本运营管理机制，完善国有企业经营业绩考核。三是大力发展非公有制经济。坚持崇尚创新、宽容失败、支持冒险、鼓励冒尖，营造尊重、爱护、宽容、保护企业家的浓厚氛围，支持民营经济发展。四是深化财税体制改革。加强税收和非税收入征管。强化财政预算绩效管理，健全覆盖各级财政的预算执行动态管理体系，加强对区级财政支出的审核管理，全面实施市直机关会计代理核算制度。对政府投资项目的中介服务实行集中招标，降低费用。五是积极探索人口管理体制机制改革。推进户籍管理向常住人口管理转变，建立人口综合信息管理系统，形成人口统筹管理大格局。六是实施“企业一套表”统计改革，完善统计制度和体系。

提高对外开放水平。优化经济技术开发区、鸠江开发区空间布局和功能定位，推进新芜经济开发区等省级开发区扩区升级，加快高沟、孙村、许镇等新批开发区筹建进度，强化综合配套，增强服务功能。推进挑商选资和精准招商，围绕世界500强、民营企业100强和行业领军企业，以战略性新兴产业和现代服务业为重点，着力引进一批投资规模大、技术含量高、带动能力强的大企业大项目。加强招商引资服务体系建设，建立重点企业“绿卡”制度。启动产业新城规划建设，在国家级和省级开发区周边高起点建设廉租房、公租房，计划5年建设1000万平方米，并完善商业、学校、医院、公交等公共服务设施，优化园区配套环境，努力把更多的农民和外来务工人员转变成安居乐业的市民，加快工业化和城市化进程。加强与长三角、珠三角地区和亳州、马鞍山等城市的区域合作，积极主动服务省江北产业集中区发展。实施市场多元化战略，扩大对外贸易，积极利用外国政府和国际组织贷款，支持企业“走出去”发展，在更高层次、更宽领域参与国际分工和合作。

四、大力加强社会建设和管理

加强社会建设和管理，是科学发展、全面转型最基本的内在要求。今年安排投入49亿元，实施39项民生工程，推进基本公共服务均等化，促进经济社会协调发展。

（一）着力实施创业富民行动

推进小额担保贷款工作，重点扶持高校毕业生、农村转移劳动力和城镇就业困难人员自主创业。加快推进国家级创业型城市创建工作，大力实施“创业扶持工程”、“大学生创业引领计划”，以创业带动就业。加强职业技能培训，完善职业技能鉴定体系，实施新技师培养计划，培育高技能人才。做好城乡统筹就业全国试点工作，加强城乡公共就业服务，推进就业服务和管理信息化，拓宽就业渠道，实施困难群体就业援助。健全劳动关系协调机制，推行工资集体协商制度，维护劳资双方合法权益。积极落实事业单位收入分配制度改革任务、离退休人员待遇调整政策。加大对群众财产权益的保护力度，创造条件让更多群众拥有财产性收入。

（二）完善覆盖城乡居民的社会保障体系

完善养老、医疗、失业、工伤、生育保险政策，扩大社保覆盖面，提高保障待遇。全面实施城乡居民养老保险，健全城镇居民医疗保险门诊统筹制度，完善城镇职工医疗保险费用支付结算办法。强化对定点医疗机构、零售药店的监管，规范医疗保险服务行为。建立工伤预防、补偿、康复制度体系。健全城乡居民最低生活保障制度，坚持困难群众价格补贴制度，加强社会救助体系建设，发展慈善事业。加强住房保障，完善宜居集团保障房融资、建设、管理机制，开工建设公租房、廉租房6.1万套，竣工保障性住房7000套，完成棚户区改造7700户，新增租金补贴1300户，对低收入住房困难家庭应保尽保。加快推进社会保障卡发放，实现社保服务“一卡通”。

（三）加快发展社会事业

推进教育均衡，保障教育公平。优化城乡中小学布局，开工建设东区义务教育阶段学校、三山中学新校区。加快学前教育、职业教育和民办教育发展，新增公办幼儿园10所、普惠性幼儿园52所，建成工业学校。支持在芜高校建设，发挥高校促进地方发展的重要作用。加强科普工作，提高公众科学素养。健全公共卫生和医疗服务体系，完成县级医院标准化建设。加快奥园二期项目等体育基础设施建设，促进全民健身，发展体育产业。稳定低生育水平，加强流动人口计划生育服务管理和出生人口性别比综合治理，开展出生缺陷干预。做好民族、宗教工作，保障妇女、儿童合法权益，完善养老服务体系，支持残疾人事业发展。

（四）维护社会和谐稳定

完善党委领导、政府负责、社会协同、公众参与的社会管理格局，不断提高社会管理科学化水平。推进基层管理体制机制改革，提高基层干部和工作人员薪资、社保待遇，探索社区治理新模式，加强和谐社区建设，做好新一届居委会换届选举工作，推动公益慈善、社会服务等社会组织健康发展。健全互联网管理体系，发挥“市民心声”等平台作用，促进政民互动。做好流动人口和特殊人群服务管理。推进依法治市，增强全社会法制意识，规范法律服务市场，提升法律服务水平。完善社会矛盾调解机制，畅通群众诉求表达渠道，规范信访秩序，维护群众合法权益。坚持和完善社会稳定风险评估机制，从源头预防和化解社会矛盾。完善食品药品安全监管体制，提高食品药品安全和质量保障水平。落实安全生产责任制，加大隐患排查治理力度，对隐患整改不达标的企业一律实施关停。健全综合防灾减灾体系，完善突发事件应急管理体制，提高危机管理和风险管理能力。推进“双拥”共建、国防建设和民兵预备役工作。加强社会治安综合治理，严厉打击违法犯罪行为，不断提升群众安全感和满意度，建设平安芜湖。

五、奋力建设文化强市

文化是城市的灵魂，是城市综合竞争力的重要体现。我们必须深入贯彻党的十七届六中全会精神，强化文化自觉和文化自信，以更大力度推进文化改革发展，建设文化强市。

（一）加强社会主义核心价值体系建设

大力弘扬以爱国主义为核心的民族精神和以改革创新为核心的时代精神，弘扬“开放、诚信、务实、创新”的城市精神，弘扬创新文化、和谐文化、诚信文化、廉政文化，深入推进社会公德、职业道德、家庭美德、个人品德建设。以争创全国文明城市为目标，努力提升市民文明素质。推进政府诚信、商务诚信、个人诚信、社会诚信体系建设，建立健全覆盖全社会的征信系统。坚持正确的舆论导向，弘扬真、善、美，针砭假、恶、丑，形成积极进取、崇尚文明的良好社会环境。

（二）提高公共文化服务水平

牢固树立文化民生理念，完善文化事业发展规划，健全公共文化服务体系，推动公共文化服务向基层倾斜、向农村延伸、向公众开放。加快市博物馆、规划馆等重点文化工程建设，实施县级文化馆和图书馆、街道文化站、社区文化服务中心、村文化活动室及文化信息资源共享工程服务点等文化惠民工程，推进全国公共电子阅览室建设试点，办好文化旅游节等节庆活动。加大公共文化投入，鼓励民营资本投资文化事业。生动展示芜湖人民创造历史的辉煌业绩，打造思想性、艺术性、观赏性相统一的精品力作。大力发展黄梅戏、梨簧戏、庐剧、铁画、剔墨纱灯等地方文化艺术，深入发掘历史文化底蕴，保护利用文化遗产，打造芜湖特色文化品牌。

（三）大力发展文化产业

完善文化产业发展规划和扶持政策，推动文化产业与现代科技深度融合，重点发展创意设计、数字动漫、影视传媒、演艺演出、文化旅游业，培育文化产业集群，打造具有国际影响力的动漫之都和文化产业集聚区。坚持重大项目带动，推进芜湖（鸠江）文化创意产业园、华仑国际文化广场、芜湖古城、利海文化生态园等项目建设，建成安徽文化戏曲公园（严凤英大舞台）、滨江文化艺术中心，引进一批重点文化产业项目。加强文化产业基地规划，推进国家动漫产业基地建设，力争文化科技产业示范区成为国家级文化和科技融合示范基地。引导社会资本投资文化产业。加强文化市场监管，营造公平竞争环境。扩大文化消费，加快数字影院建设，争创国家数字出版基地，新建大型数字出版物批发市场。加强对外文化交流，鼓励文化“走出去”。

六、着力加强政府自身建设

坚持以人为本、依法治市，打造勤政爱民、依法行政、廉洁高效的务实政府。

（一）坚持依法行政。深入贯彻依法行政实施纲要，加快法治政府建设。自觉接受人大监督、政协民主监督，以提高人大议案、代表建议和政协提案的办理质量推进政府相关工作。认真听取吸纳各民主党派、工商联、无党派人士和各人民团体的意见建议。完善行政决策程序，推进决策科学化、民主化，强化重大决策跟踪反馈和责任追究。完善政府管理制度，加强对规范性文件的审查和清理。落实行政执法责任制，推行行政处罚自由裁量权基准制度，规范行政执法行为。

（二）提高行政效能。深入开展机关效能建设活动，以增强政府机关工作人员服务科学发展能力为总要求，大力倡导各项工作都要用心、用情、用力，坚决治庸、治懒、治散。加强行政服务中心建设，推行服务质量公开承诺制。坚持政务公开，强化网络问政，主动接受人民群众和社会舆论监督。深化行政审批制度改革，加强电子政务建设，实行网上公开申报、受理、咨询和办复，提高办事效率，强化便民服务。

（三）加强廉政建设。大力推进惩治和预防腐败体系建设，加大从源头上防治腐败的工作力度。深入开展纠风工作，坚持万人千企评议政风行风，坚决纠正损害群众利益的不正之风。加强工程建设招投标、土地使用权“招拍挂”、产权交易、政府采购等重点领域的监督检查，对腐败案件坚决查处，对腐败分子坚决严惩。加强财政收支预算管理，“紧”行政支出，“松”民生和新兴产业投入。充分发挥审计监督职能，坚持政府性资金审计全覆盖。加强政府内部层级监督，严格行政问责，坚决查处失职渎职、行政不作为等行为，营造风清气正、昂扬向上的良好政务环境。

各位代表！

过去一年的发展成就令人鼓舞，新的一年的美好前景催人奋进。让我们在中共芜湖市委的坚强领导下，倍加珍惜芜湖发展的难得机遇，倍加珍惜来之不易的崛起势头，倍加珍惜精诚团结的浓厚氛围，倍加珍惜和谐稳定的大好形势，保持科学发展的激情，坚定率先崛起的信心，倍道兼程，奋发有为，开创新局面，实现新跨越，以优异成绩迎接党的十八大胜利召开！

附：部分名词解释

1.行政区划调整：2011年8月22日我省部分行政区划调整后，芜湖市域面积由3317平方公里扩大到5988平方公里，户籍人口由229.5万增加到384.2万；市区面积由826.7平方公里扩大到1064.7平方公里、人口由111.5万增加到123.8万。

2.企业股权和分红激励：这是高新技术产业发展过程中的一项重大制度创新。合芜蚌试验区正式加入北京中关村、武汉东湖、上海张江这三个国家自主创新示范区股权和分红激励试点序列，享受同等政策待遇。试验区内科技创新型企业，可将3年税后利润形成的净资产增值额的35%，以股权奖励、股权出售、股票期权等形式对企业有突出贡献的技术人员和经营管理人员实施激励。试验区企业自行或联合转化科技成果，以及高校、科研院所以科技成果作价入股企业等，科技人员都将获得一定比例的收益。

3.“新三板”：特指中关村科技园区非上市股份有限公司进入代办股份系统进行转让试点。挂牌企业均为高科技企业，不同于原转让系统内的退市企业及原STAQ（全国证券交易自动报价系统）、NET系统（中国证券交易系统有限公司开发设计）挂牌公司，故形象地称为“新三板”。

4.“企业一套表”：是以统计调查对象为核心，通过整合现行统计报表制度，规范统计标准和业务流程，在同一软件系统下采集、审核、上报、汇总和处理统计数据的一种新型统计调查制度。
